# Does the Spatial Layout of a Playground Affect the Play Activities in Young Children? A Pilot Study

**DOI:** 10.3389/fpsyg.2021.627052

**Published:** 2021-05-28

**Authors:** Masashi Sumiya, Tetsushi Nonaka

**Affiliations:** ^1^Department of Human Studies, Seisen University, Hikone, Japan; ^2^Graduate School of Human Development and Environment, Kobe University, Kobe, Japan

**Keywords:** playground, spatial layout, free play, young children, accelerometer, physical activity, play equipment, outdoor play

## Abstract

**Background:**

The objective of this study was to describe, through measurement of physical activity and observation of free outdoor play, the relation between children’s free play and the spatial layout of the playground. To accomplish this, we altered the spatial layout of the same playground to see how the layout affects the play activity and the physical activity levels in the same children.

**Methods:**

Participants were six young children (four girls and two boys; mean age = 5 years and 1 month, SD = 2.59 months). Participants’ physical activity level and the duration of different types of action that occurred in each area and their transitions were compared before and after the alteration of the play-equipment layout using the data from accelerometers and video recordings.

**Results:**

A significant increase in physical activity occurred after the spatial layout alteration, which was related to action differences. Before the alteration, children tended to play in a similar manner for a given play area; however, after the alteration, pronounced interindividual variation in play activity across children was observed.

**Conclusion:**

The present pilot study found that in free play situations in the outdoor playground, the spatial layout of playground affects the pattern of play activity and the physical activity levels of young children.

## Introduction

Over the past 50 years, play opportunities for children, especially free play in outside play spaces, have continually declined in some countries (Burdette and Whitaker, 2005; Malone, 2007). This trend contrasts the research-grounded understanding of the importance of play for children’s healthy development (Ginsburg, 2007; Pellegrini et al., 2007). The decline in the opportunities for free play makes preschool playgrounds all the more important, where young children spend an ample amount of time and can choose what to do freely. Free play is an activity that is freely chosen and is performed for individuals’ own sake. Adult-directed structured play, such as games and sports, do not fall into the category of free play (Burdette and Whitaker, 2005; Gray, 2011). Through play, children can experiment, solve problems, think creatively, corporate with others, and gain a deeper knowledge about themselves and the world. Today, it is crucial that we better understand the nature of children’s free play behaviors and how we can promote them.

Play activities have been quantitatively described in terms of physical activity levels as measured by step counts or accelerometers. Previous studies found that physical activity levels are influenced by individual or environmental factors. The individual factors included sex (Hinkley et al., 2008), ethnicity (Sallis et al., 1993), and fundamental movement skills (Fisher et al., 2005; Williams et al., 2008; Cohen et al., 2014), while the environmental factors included parents’ obesity (Danner et al., 1991), parents’ physical activity level (Xu et al., 2018), active or outdoor playtime (Burdette et al., 2004; Raustorp et al., 2012), the amount of play equipment (Herrington and Lesmeister, 2006; Sando and Sandseter, 2020), the number of children per m^2^ in the playground (Cardon et al., 2008; Van Cauwenberghe et al., 2012), playground esthetics or safety (Farley et al., 2007; Gray, 2011), natural play area (Fjørtoft and Sageie, 2000; Dowdell et al., 2011; Herrington and Brussoni, 2015), spatial layout of equipment (Liempd et al., 2020), institutional policy (Dowda et al., 2009; Trost et al., 2010), physical activity training and education for teachers and/or parents (Bower et al., 2008), and sociocultural differences (Leeger-Aschmann et al., 2016).

In preschools, various intervention methods to promote spontaneous free play have been proposed, which include decreasing playground density (Van Cauwenberghe et al., 2012), the provision of play equipment (Hannon and Brown, 2008), the provision of playground markings (Cardon et al., 2009), teachers’ encouragement (Brown et al., 2009), and increased recess duration (Holmes et al., 2006). A couple of methods, such as decreasing playground density and provision of portable play equipment, resulted in partially increased physical activity (Hannon and Brown, 2008; Van Cauwenberghe et al., 2012), and another study reported positive cognitive effects (Holmes et al., 2006). However, there are also a number of other studies that have reported inconclusive evidence concerning the causal relationship between playground features and children’s play activity pattern or physical activity level (Mota et al., 2005; Ridgers and Stratton, 2005; Stratton and Mullan, 2005; Ridgers et al., 2007; Bohn-Goldbaum et al., 2013; Broekhuizen et al., 2014).

In the present study, we altered the spatial layout of the same playground to see how the layout affects the play activity and the physical activity levels in the same children. The central concept guiding this alteration of spatial layout is that of affordances. Gibson (1979) developed this concept to account for the fact that humans and animals including young children primarily pay attention to and perceive what they can do in a given environment, instead of perceiving the abstract qualities of objects in the environment such as position, shape, and color as such (Gibson, 1979). Young children’s play is always situated or embedded (Adolph et al., 2019), and the possibilities offered by the environment are the basic conditions for spontaneous play. In order to promote children’s free play in the playground, it is desirable that the playground has a rich set of affordances that afford a wide variety of actions by young children.

Based on the concept of affordances, in the present study, we aimed to enrich the variety of play activities of children by altering the spatial layout of the playground in a daycare center in Japan. After the discussion with caregivers in the daycare center, we altered the playground based on the following three basic plans: (1) to make available the affordance for climbing (e.g., a climbable mound), (2) to afford easy access to toys and objects used in each area, and (3) to clearly separate play areas with a different set of affordances. Since the alteration of the playground was conducted in an exploratory manner, in the present study, we focused on general questions such as the following: Does the alteration of the spatial layout of playground affects the pattern of play activity of young children playing in the playground? Does the alteration of the spatial layout of playground affects the physical activity levels of young children playing in the playground?

## Materials and Methods

### Participants

Six children (four girls and two boys; *M* = 5 years and 1 month, SD = 2.59 months) from 4-year-old classes at a private certified daycare center in Osaka, Japan participated. All participants voluntarily participated in this study. During the observations, all children from other classes were also playing in the playground. The daycare center was in its third year and had a capacity of 90 children, aged 0–6 years, when the present observations took place. Informed consent forms were obtained from all children’s parents and the principal of the daycare center.

### Playground

**FIGURE 1:**
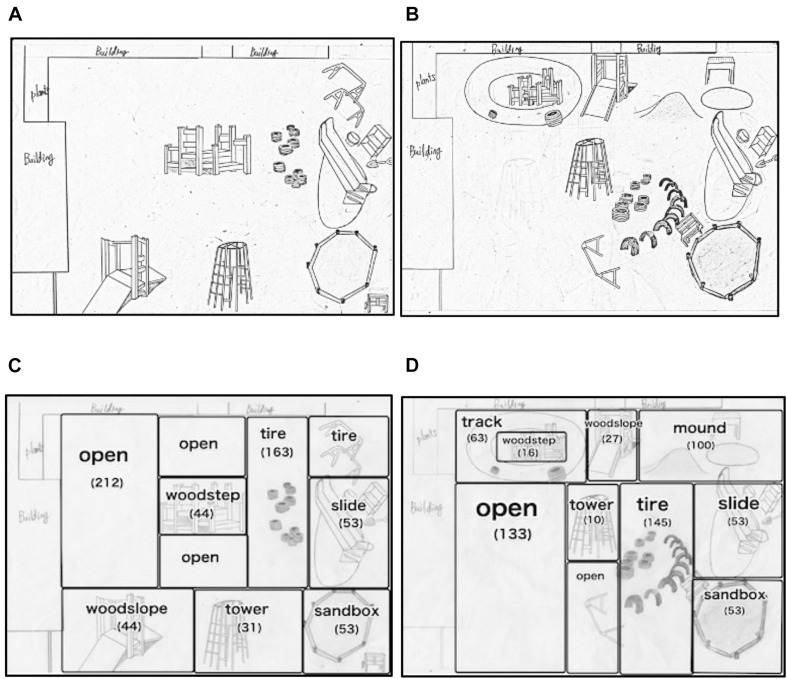
Illustration of the spatial layout of the playground and the area separations for observation. **(A,B)** illustrate the spatial layout before and after the alteration, respectively. **(C,D)** indicate area separations before and after the alteration, respectively. The numbers indicated below the area name are the square meters (m^2^) of the area.

The original spatial layout of the playground of the daycare center is illustrated in Figure 1A. The playground was segmented into 11 areas indicated in Figure 1C: 10 areas surrounding each play equipment and an area of open space. The detailed features of play equipment are indicated in Table 1. The total ground area was about 600 m^2^. The sandbox and slide were fixed on the opposite side of the building near a fence (illustrated in the lower right part of Figure 1A). Utensils used for sand play were placed on tool shelves near the slide area by the fence. At the center of the playground, between the open and tires areas, there was play equipment made of wood with steps that afforded climbing (referred to as “woodstep,” see Table 1). Ten tires were unevenly placed in the part of the open space (referred to as “tires,” see Table 1). There was a slope made of wood (referred to as “woodslope,” see Table 1) and a tower-shaped climbing frame (referred to as “tower,” see Table 1) next to the sandbox along the fence.

**TABLE 1:** The detailed features of the play equipment and area.

Area	Feature(s)
Sandbox	The ground area of the sandbox covered with sand was about 17 m^2^, framed with 10-cm-diameter logs. There was wooden kitchen-like play equipment about 130 cm tall.
Slide	The slide had no stairs; it was fixed on a mound with the top of the slide about 1 m off the ground.
Tires	There were tires with a variety of sizes.
Open	This was a spacious area around the woodstep before the alteration. This area was segmented by lines marked on the ground, and by a movable iron bar and the tower after the alteration. There was nothing in the area after the alteration.
Woodstep	This had three levels of steps, with each level being about 20-cm high. The handrail on the highest level was 130 cm off the ground.
Woodslope	This was about 70 cm high at the top of the slope, and the top of the wooden frame was about 180 cm off the ground.
Tower	The highest part of the tower was about 190 cm, but the highest part a child could climb to was about 150 cm. After the alteration, the tower was put in the center of playground because children could climb up and observe the other children playing from above.
Mound	A handmade mound about 40-cm tall.
Track	This was marked around the woodstep after the alteration. The arrows, which indicate the running direction, were marked on the ground.

After the video observation of children’s play at the aforementioned original playground, we changed the spatial layout of the play equipment based on the discussion with the caregivers of the daycare center. By changing the spatial layout of the play equipment, we aimed to enrich the variety of play activities of children. To achieve this aim, although our approach was admittedly exploratory, we chose to alter the playground layout according to the following three plans: (1) to make available the affordance for climbing (e.g., a climbable mound), (2) to afford easy access to toys and objects used in each area, and (3) to clearly separate play areas with a different set of affordances. First point was considered because the original playground was relatively level, and there was little chance for children to explore convexities or concavities. Second point reflects the problem the caregivers were aware of in the original playground, especially in the sandbox area, in which a shelf for toys and utensils cannot be easily accessed by children while playing in the area. The shelf for utensils was located far from the sandbox, and children needed to move back and forth to use the utensils. Third point concerns safety. In the original layout, the woodstep was in the middle of the open area, and the woodslope was facing the open area. Since children played with balls mainly in the open area, balls often got in the way of play activity at those contiguous areas; thus, children playing with balls were somewhat restricted, while other children felt uneasy that they might be hit by balls.

The spatial layout was altered following the above basic plans (see Figures 1B,D). First, a mound was created in an area on the side of the building where tires had been scattered before the alteration, and 10 tires were embedded in the ground vertically in the remaining area of the original tires area (see Table 1). The tower was placed at the center of the playground. The woodstep was placed near the building, and a track was created around it by marking a line (see Table 1). Children could climb on and sit or stand on the tower or the woodstep and observe other children from the height. Second, we placed play equipment between the sandbox and tires areas to provide various play opportunities in the sandbox area. Some of the utensils were placed on the play equipment. Third, for ball play to be confined within the open area without disturbing the children playing in the other areas, the open area was segmented with play equipment or lines, which were marked on the ground to create the aforementioned track. There was no play equipment in the open area. With the segmentation, the open area became a separated, rectangular area.

### Measurement and Observation Procedures

#### Measurement of Physical Activity

Children’s physical activity level was measured with an Actigraph wGT3X accelerometer (Actigraph Corp., Pensacola, FL, United States). Actigraph wGT3X is a lightweight (19 g; 4.6 cm × 3.3 cm × 1.5 cm), triaxial accelerometer, which collects motion data on three orthogonal axes—vertical (*Y*), horizontal right–left (*X*), and horizontal front–back axis (*Z*)—between 0.05 and 2.0 Gs. This accelerometer has been validated for use with children in laboratory and field settings (Santos-Lozano et al., 2012; Delisle et al., 2015; Carson et al., 2019). Participants wore this equipment using an elastic belt at the waist. The analog acceleration data were converted to a digital signal, and the value (count) was stored in specified time interval (epoch). Ten-second epochs were used for this study. After data collection, the monitor was downloaded to a computer for subsequent data reduction and analysis.

#### Video Recording

The free play in the playground was recorded on November 27, 2018 (referred to as “before the alteration”) and on January 23, 2019 (referred to as “after the alteration”). The alteration of the spatial layout of the playground was implemented on December 16, 2018. Video recording was performed with three cameras (Sony HDR-PJ675), which were set to record most of the playground. The climate of the area was warm, and the temperature on the day of observation was 19°C for before the alteration and 16°C after the alteration. All participants wore long sleeve T-shirts and sometimes rolled up their sleeves. At the center that we observed, children usually have free, unstructured outdoor play time from around 10:30 A.M. to 12:00 P.M., without any instructions from caregivers. The observation time was 97 min for before the alteration and 87 min for after the alteration.

#### Action Coding

The actions that appeared during play were coded by observing the video recordings (Adolph, 2016). Table 2 summarizes the code names and definitions. Ten categories, which included (1) locomotion, (2) climbing, (3) manipulation, (4) sedentary, (5) cycling, (6) sloping, (7) sand play, (8) water play, (9) sporting, and (10) horseplay, were created during observation. Categories were defined so that multiple codes would not overlap and so children’s actions could be described without leaving anything out. The coding was performed using video analysis software (Datavyu^1^). We recorded the times when participants (1) started and ended the actions and (2) entered and exited the areas. The first coder coded 100% of children’s actions; then, a second coder coded 25% of the total time. The coding agreement rate for the behavior categories was 0.91.

**TABLE 2:** Children’s actions observed in play were coded into these 10 categories.

Code	Definition
Locomotion	Transition between two places (e.g., walk, run, skip, and step across tires)
Climbing	Vertical climbing up and down (e.g., climbing up or down on the tower, climbing up on the top of the woodslope handrails, or jumping down from the top of woodstep handrail)
Manipulation	Using detached objects (e.g., balls and hula hoop)
Sedentary	Quiet, small amount of physical movement (e.g., chatting with others, doing nothing, sitting, or looking down or sitting on the tower)
Cycling	Play with any kind of vehicle (e.g., tricycle, bicycle, kickboard, and buggy)
Sloping	Play with any kind of slope (e.g., running up the slide, cycling down from the top of the mound, and sliding down the slide)
Sand play	Using sand (including touching sand or drawing something on the ground)
Water play	Using water (including carrying water with buckets or using a watering can)
Sporting	Sporting games that include commonly understood rules and were freely chosen by children
Horseplay	Playful physical contact or fawn (e.g., pretend-hero play and playful pushing)

### Data Analysis

Data analyses were conducted as follows. Participants’ physical activity level was modeled using a linear mixed model with fixed-effects for layout (original vs. altered) and area (nine areas) factors and random intercept effects for individual children. To investigate the duration of actions in each area, the proportion of duration of observed actions in each area (referred to as “assemblage of actions”) were calculated for the original playground and after the alteration of layout. A multivariate non-parametric permutational analysis of variance was performed to test the presence of significant variations in assemblage of actions among areas and among individuals using the adonis function in R package vegan (Oksanen et al., 2016). We constructed dendrograms to visualize the similarities among the assemblage of actions of each individual play episode according to the unweighted pair-group mean arithmetic (UPGMA) method using R function hclust. To examine the visiting sequences of transitions among different areas in the playground, we summarized the sequential relationships between areas in the playground visited by children in a transition matrix—separately for before and after the alteration of spatial layout. A Chi-square test of quasi-independence was used to test whether the overall sequential pattern differed from a random ordering of events. Binomial test *z*-scores (adjusted residuals) were computed from frequency counts of transitions between each consecutive behavior corresponding significance levels (Bakeman and Quera, 2011). Significance level for statistical analysis was set at 0.05.

## Results

Children’s playing time was 83 min 24 s before the alteration and 62 min 18 s after the alteration (see Table 3). In particular, for the play times, after the alteration, three participants—P2, P4, and P6—were under 60 min because of class activities. In the original playground, four children spent most of their time at the sandbox area, and two were most often at the woodslope area and playing with sand. In the altered playground, children spent most of their time at various places. P1 was mostly in the open area, P3 and P4 were at the sandbox area for more than 20 min, and P5 and P6 were at the tires area for around 40 min.

**TABLE 3:** Participants’ playing time: total and in each area.

	P1	P2	P3	P4	P5	P6	Total
**(A) Before the alteration**							
Sandbox	2.2	38.2	17.5	49.8	36.6	35.5	179.8
	3%	47%	20%	57%	46%	43%	36%
Slide	2.0	11.4	4.2	2.2	0.2	5.2	25.2
	2%	14%	5%	3%	0%	6%	5%
Tires	7.5	9.1	9.6	5.8	8.0	8.6	48.6
	9%	11%	11%	7%	10%	10%	10%
Open	21.4	9.5	8.6	16.5	13.6	18.1	87.6
	25%	12%	10%	19%	17%	22%	18%
Woodstep	5.3	5.3	5.5	1.8	4.2	1.1	23.1
	6%	7%	6%	2%	5%	1%	5%
Woodslope	33.3	–	36.5	5.2	0.7	1.3	77.0
	40%	–	42%	6%	1%	2%	15%
Tower	12.3	7.6	4.7	5.7	16.2	12.4	58.9
	15%	9%	5%	7%	20%	15%	12%
Total	84.1	81.1	86.6	86.9	79.4	82.1	500.2
**(B) After the alteration**							
Sandbox	–	2.8	33.0	21.1	1.4	1.3	59.5
	–	8%	41%	41%	2%	2%	16%
Slide	–	1.8	5.4	8.5	0.6	2.2	18.4
	–	5%	7%	17%	1%	4%	5%
Tires	–	10.5	2.8	1.1	41.9	38.5	94.7
	–	29%	3%	2%	57%	65%	25%
Open	72.4	2.4	6.8	2.2	3.6	4.9	92.4
	98%	7%	8%	4%	5%	8%	25%
Woodstep	1.1	1.2	3.1	2.6	3.7	0.5	12.1
	1%	3%	4%	5%	5%	1%	3%
Woodslope	–	0.4	0.7	0.2	–	4.5	5.9
	–	1%	1%	0%	–	8%	2%
Tower	–	5.6	4.2	0.8	12.9	0.2	23.6
	–	16%	5%	1%	17%	0%	6%
Mound	–	9.7	21.8	12.6	5.0	4.7	53.7
	–	27%	27%	25%	7%	8%	14%
Track	0.3	1.3	2.4	2.1	4.7	2.6	13.5
	0%	4%	3%	4%	6%	4%	4%
Total	73.8	35.6	80.0	51.2	73.9	59.3	373.7

*The upper row indicates minutes playing at each area, and the lower row indicates the ratio of playing time at each area to total playing time.*

*P, participant.*

### Assemblage of Actions in Each Area

**FIGURE 2:**
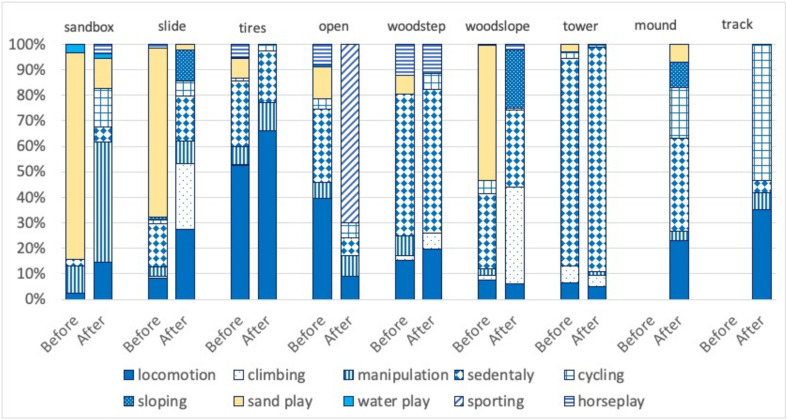
Comparison of assemblage of actions before and after the spatial alteration. The data for mound and track areas were not available before the alteration.

Figure 2 presents the proportion of duration of 10 categories of action (Table 2) observed in each area of the playground before and after the alteration of the layout. The proportion of actions observed during play in each area differed between the original and altered spatial layout. For example, the relative duration of manipulation increased in the sandbox area and that of locomotion, sloping, and climbing increased in the slide area, respectively. The proportion of sporting increased in the open area and that of sloping and climbing increased in the woodslope area, respectively (Figure 2). On the other hand, the relative duration of sand play generally decreased. In the newly constructed mound area, sedentary occupied the highest percentage at 37%, and locomotion and cycling were 23 and 20%, respectively. In the track area, cycling was the most popular action (53%), and locomotion was the second (35%) (Figure 2).

**FIGURE 3:**
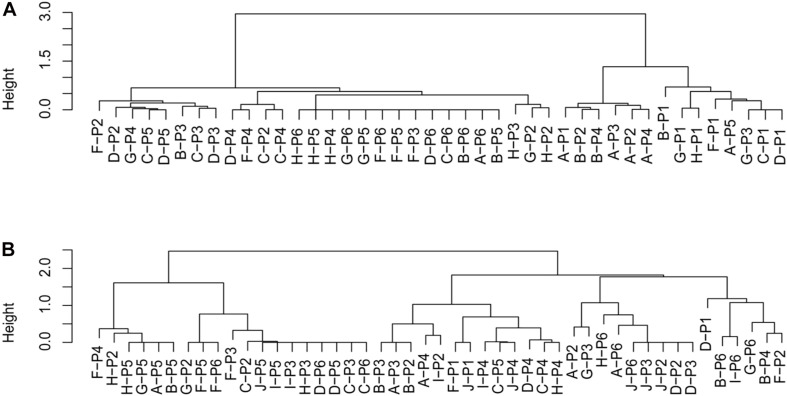
The hierarchical clusters of duration of each action in each area before and after the alteration [**(A)** dendrogram of before the alteration, **(B)** dendrogram of after the alteration]. The height, shown on the *y*-axis, means the similarity between action durations in the given area. The letters in dendrograms indicate each area (A, sandbox; B, slide; C, tires; D, open; F, woodstep; G, woodslope; H, tower; I, mound; and J, track).

We constructed dendrograms to visualize the similarities among the assemblage of actions of each individual play episode. The dendrograms are shown in Figure 3A for before the alteration and Figure 3B for after the alteration. In the dendrogram for before the alteration (Figure 3A), small clusters of H (tower), F (woodstep), and A (sandbox) are observed. By contrast, in the dendrogram for after the alteration of the layout (Figure 3B), except J (track), clustering based on areas disappeared. Instead, clustering of the same participant (notably P5, P4, and P6) emerged. To statistically examine the effect of area and participant on the proportion of actions of play episode, non-parametric permutational multivariate analyses of variance showed that, before the alteration, the Area had a significant effect on the assemblage of play action. By contrast, after the alteration, a significant effect from the Area was not detected; however, the Individual did have a significant effect (see Table 4).

**TABLE 4:** Permutational multivariate analysis of variance results for the assemblage of the 10 action categories observed during play [*R*^2^ (coefficient of determination) indicates how well each factor explains the dependent variable].

	Factor	*df*	*R*^2^	*p*
Before alteration	Area	6	0.48	0.0002
	Individual	5	0.03	0.8655
	Residuals	30	0.49	
After alteration	Area	8	0.13	0.412
	Individual	5	0.36	0.0003
	Residuals	33	0.51	

### Physical Activity Measurement

**FIGURE 4:**
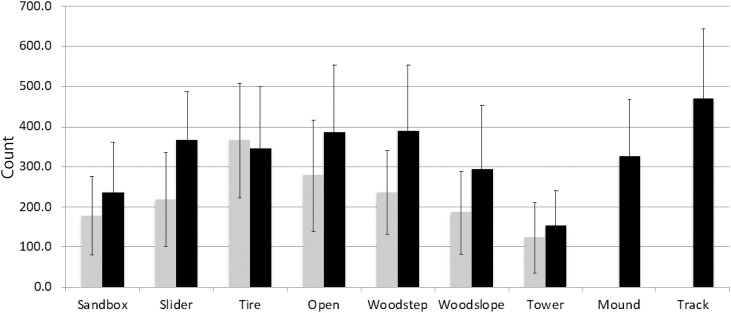
The averaged accelerometer counts per epoch before and after the alteration. The data for mound and track areas were not available before the alteration.

The average counts of all participants per epoch that reflect the children’s physical activity levels are shown in Figure 4. Linear mixed model analyses on the children’s physical activity levels with fixed-effects for area and layout and random intercept effects for individual children revealed significant effects of both Layout, *F*(1,69) = 16.88, *p* < 0.001, and Area, *F*(8,66) = 2.91, *p* = 0.008, but no significant interaction was found between the two factors. Bonferroni-corrected multiple comparisons revealed that the children’s physical activity levels were significantly greater in the tires, open, and track areas as compared to the tower area (*p*s < 0.05). Children’s physical activity levels after the alteration were significantly greater in the sandbox, open, woodstep, and woodslope areas.

### Transitions Between Places

To investigate the visiting sequences of transitions among different places in the playground, we summarized the sequential relationships between places in the playground visited by children in a transition matrix, separately for before and after the alteration (see Table 5). In a chi-square test of quasi-independence, before the alteration, the overall sequential pattern differed significantly from a random ordering of events, χ^2^(29) = 124.43, *p* < 0.001. However, after the alteration, the overall sequential pattern was not significantly different from a random ordering of events, χ^2^(55) = 64.63, *p* = 0.18), indicating that there were no stereotyped patterns of sequential transitions between places in the playground. We further calculated binomial test *z*-scores (adjusted residuals) from frequency counts of transitions between each consecutive behavior corresponding significance levels. In the original playground, the transition between the sandbox and tower and the transition between the tires and open areas occurred much more frequently than expected in both directions, *p* < 0.001. In contrast, after the alteration, no such clear tendency of sequential transition patterns was observed except that unidirectional transitions were more likely to occur than chance from open to sandbox and, to a lesser degree, from sandbox to slide.

**TABLE 5:** Frequencies of sequential transition between consecutive places in playground before and after the alteration.

(A) Before the alteration									

	Following place								
	
Preceding place	Sandbox	Slide	Tires	Open	Woodstep	Woodslope	Tower		
Sandbox	–	3**	1	0^††^	0	0	5**		
Slide	1	–	8	0^††^	0	0	2		
Tires	0^†^	8	–	34**	7	7	0^††^		
Open	0	0^†^	19*	–	4	5	2		
Woodstep	0	0	3	8	–	0	0		
Woodslope	0	0	3	6	0	–	3		
Tower	7**	1	2	1^†^	0	1	–		

**(B) After the alteration**									

	**Following place**								
	
**Preceding place**	**Sandbox**	**Slide**	**Tires**	**Open**	**Woodstep**	**Woodslope**	**Tower**	**Mound**	**Track**

Sandbox	–	6*	3	2	1	0	1	3	0
Slide	2	–	1	4	0	0	0	3	1
Tires	1	4	–	5	0	0	0	6	1
Open	8**	1	4	–	2	0	0	5	3
Woodstep	0	0	0	0	–	0	0	2	1
woodslope	0	0	1	0	1	–	1	0	1
Tower	0	0	0	0	1	0	–	1	0
Mound	3	5	4	3	1	0	0	–	4
Track	0	1	4	2	1	1	1	2	–

*Observed > Expected, *p* < 0.05.

**O > E, *p* < 0.01.

*^†^O < E, *p* < 0.05.*

*^††^O < E, *p* < 0.01.*

## Discussion

The present study explored the influence of the spatial arrangement of play equipment in a playground on children’s play activity patterns and physical activity levels. We aimed to enrich the variety of play activities of children by making the following changes to the spatial layout of playground: (1) to make available the affordance for climbing (e.g., a climbable mound), (2) to afford easy access to toys and objects used in each area, and (3) to clearly separate play areas with a different set of affordances. Before the alteration, children tended to play in a similar manner for a given play area; however, after the alteration, pronounced interindividual variation in play activity across children was observed. We also observed that the alteration of play equipment layout promoted new types of play such as organized, sporting play, locomotive play (e.g., walking across tires), and imitative play (e.g., using sandbox, kitchen-like play equipment, and utensils). The frequencies of sequential transition between consecutive places in the playground were also altered, indicating the reduction in stereotypical patterns of sequential transitions between places after the alteration of the playground. These changes in the play activities of children lead them to display increased level of physical activity after the alteration of the spatial layout compared to that in the original playground.

### The Relation Between Play Patterns and Physical Activity Levels

When we changed the spatial arrangement in the playground, we created the area with vertically embedded tires and the area with a climbable mound so that children can climb onto them and experience the height. The increase in the duration of free play was observed in three children in the tires area, and the duration of locomotion was increased as they played by walking on the embedded tires. The marked increase in the duration of play was also observed in two children in the mound area. We also improved the accessibility to utensils and toys in the sandbox area so that children could play with utensils, for example, to imitate cooking. As a consequence, the children moved back and forth to carry sand or to manipulate utensils rather than to sit and to mold sand as they did in the original playground. The open area was demarcated by placing other play equipment. The demarcation of the open area afforded the organized soccer play, which in turn increased physical activity levels in children played in this area.

### Implications for Play Ground Design

Although it may be difficult to install a new set of play equipment or renovate the whole playground at daycare centers or in public parks, as demonstrated in the present study, it is possible to alter the spatial layout of play equipment in such a way to influence children’s free play behavior. Previous studies reported that the altered mode of play, the reported level of fun, or the assessment of playground would be heightened through non-standardizing the design of stepping stones in older children (Prieske et al., 2015; Sporrel et al., 2017). The findings of the present study add to these evidence by showing that even young preschool-aged children spontaneously changed their play patterns in such a way to use different opportunities provided by the playground. This finding may have practical implications: although it would be difficult to have preschool-aged children verbally assess the playground (c.f., Sporrel et al., 2017), it is possible for designers to assess the play activities of young children after rearranging the equipment in a manner conducted in the present study and to further examine what kind of environment affords children’s free play.

### Limitations: Other Possible Explanation of Increased Physical Activity

The alteration of the spatial layout of the playground was an irreversible process. Once the spatial layout had been changed, it was not possible to restore the original layout of the playground. Because of this, we could not have a control group who play in the original playground twice in addition to the children who played in the altered playground. Another limitation of this study was the small sample size, which means that it may be difficult to generalize the results of this study. For the future suggestion, the larger sample size from various developmental stages or multiple single-subjects study design will be necessary. Another limitation of the present study might be the short period between the alteration of the playground and the measurement of play. We might have assessed a novelty effect, even though a month had passed since the alteration. We cannot articulate the optimal interval, but we also cannot rule it out as the reason behind the increased physical activity.

## Conclusion

The present study reported that young children’s mode of play in a playground could be altered by the spatial rearrangement of the play equipment. Young children may have perceived unique possibilities or constraints related to this alteration. Children’s mode of play is thus affected by the arrangement of the playground, and this should be explored further in intervention studies.

## Data Availability Statement

All data are available on reasonable request from the corresponding author.

## Ethics Statement

Ethical review and approval was not required for the study on human participants in accordance with the local legislation and institutional requirements. Written informed consent to participate in this study was provided by the participants’ legal guardian/next of kin.

## Author Contributions

MS and TN conceptualized and designed the study. MS acquired the data, performed the initial analysis, interpreted the data, drafted the initial manuscript, and approved the final manuscript as submitted. TN contributed to statistical analyses, interpreted the data, and critically revised the manuscript. Both authors agreed to be accountable for all aspects of the work ensuring that questions related to the accuracy or integrity of any part of the work were appropriately integrated and resolved.

## Conflict of Interest

The authors declare that the research was conducted in the absence of any commercial or financial relationships that could be construed as a potential conflict of interest.

## Footnotes

1 http://www.datavyu.org
